# Human brain patterns underlying vigilant attention: impact of sleep debt, circadian phase and attentional engagement

**DOI:** 10.1038/s41598-017-17022-9

**Published:** 2018-01-17

**Authors:** Micheline Maire, Carolin F. Reichert, Virginie Gabel, Antoine U. Viola, Christophe Phillips, Christian Berthomier, Stefan Borgwardt, Christian Cajochen, Christina Schmidt

**Affiliations:** 1 Centre for Chronobiology, Psychiatric Hospital of the University of Basel, Basel, Switzerland; 2 Institute of Primary Health Care (BIHAM), University of Bern, Bern, Switzerland; 3 Transfaculty Research Platform Molecular and Cognitive Neurosciences, University of Basel, Basel, Switzerland; 4 GIGA-CRC In Vivo Imaging, University of Liège, Liège, Belgium; 5 Medical Image Analysis Center, University Hospital of Basel, Basel, Switzerland; 6 Department of Psychiatry, University Hospital of Basel, Basel, Switzerland; 7 PHYSIP SA, Paris, France; 8 PPRS, Paris, France

**Keywords:** Circadian rhythms and sleep, Human behaviour

## Abstract

Sleepiness and cognitive function vary over the 24-h day due to circadian and sleep-wake-dependent mechanisms. However, the underlying cerebral hallmarks associated with these variations remain to be fully established. Using functional magnetic resonance imaging (fMRI), we investigated brain responses associated with circadian and homeostatic sleep-wake-driven dynamics of subjective sleepiness throughout day and night. Healthy volunteers regularly performed a psychomotor vigilance task (PVT) in the MR-scanner during a 40-h sleep deprivation (high sleep pressure) and a 40-h multiple nap protocol (low sleep pressure). When sleep deprived, arousal-promoting thalamic activation during optimal PVT performance paralleled the time course of subjective sleepiness with peaks at night and troughs on the subsequent day. Conversely, task-related cortical activation decreased when sleepiness increased as a consequence of higher sleep debt. Under low sleep pressure, we did not observe any significant temporal association between PVT-related brain activation and subjective sleepiness. Thus, a circadian modulation in brain correlates of vigilant attention was only detectable under high sleep pressure conditions. Our data indicate that circadian and sleep homeostatic processes impact on vigilant attention via specific mechanisms; mirrored in a decline of cortical resources under high sleep pressure, opposed by a subcortical “rescuing” at adverse circadian times.

## Introduction

The two-process model of circadian and homeostatic sleep-wake regulation accurately predicts human sleepiness and neurobehavioral performance over the 24-h cycle. The phase relation between the circadian pacemaker and the sleep-wake cycle is uniquely timed to maintain stable sleepiness and performance levels throughout a typical 16-h wake episode. However, extending wakefulness into the biological night is associated with steep increases in sleepiness, because the circadian pacemaker does not promote wakefulness during this time window and thereby does not counteract increasing sleep pressure levels^1^. Interestingly, when wakefulness is further extended to daytime, neurobehavioral performance partially recovers, most likely due to the reactivation of a circadian alerting signal^2,3^. While circadian and sleep loss effects on neurobehavioral performance are well established, their impact on the cerebral correlates underlying performance remain largely unknown. A recent study observed that cognitive brain responses followed circadian and homeostatic drives in a region-specific manner^4^. Furthermore, functional imaging studies on the effects of total sleep deprivation (SD) on cerebral correlates of cognitive performance indicate that sleep-loss-related decrements in performance are mirrored by decreases in task-related cortical responses (e.g.,^5–8^). In contrast, the thalamus has been identified as the only region that consistently showed increased activation as a response to sleep loss^9^.

**Figure 1:**
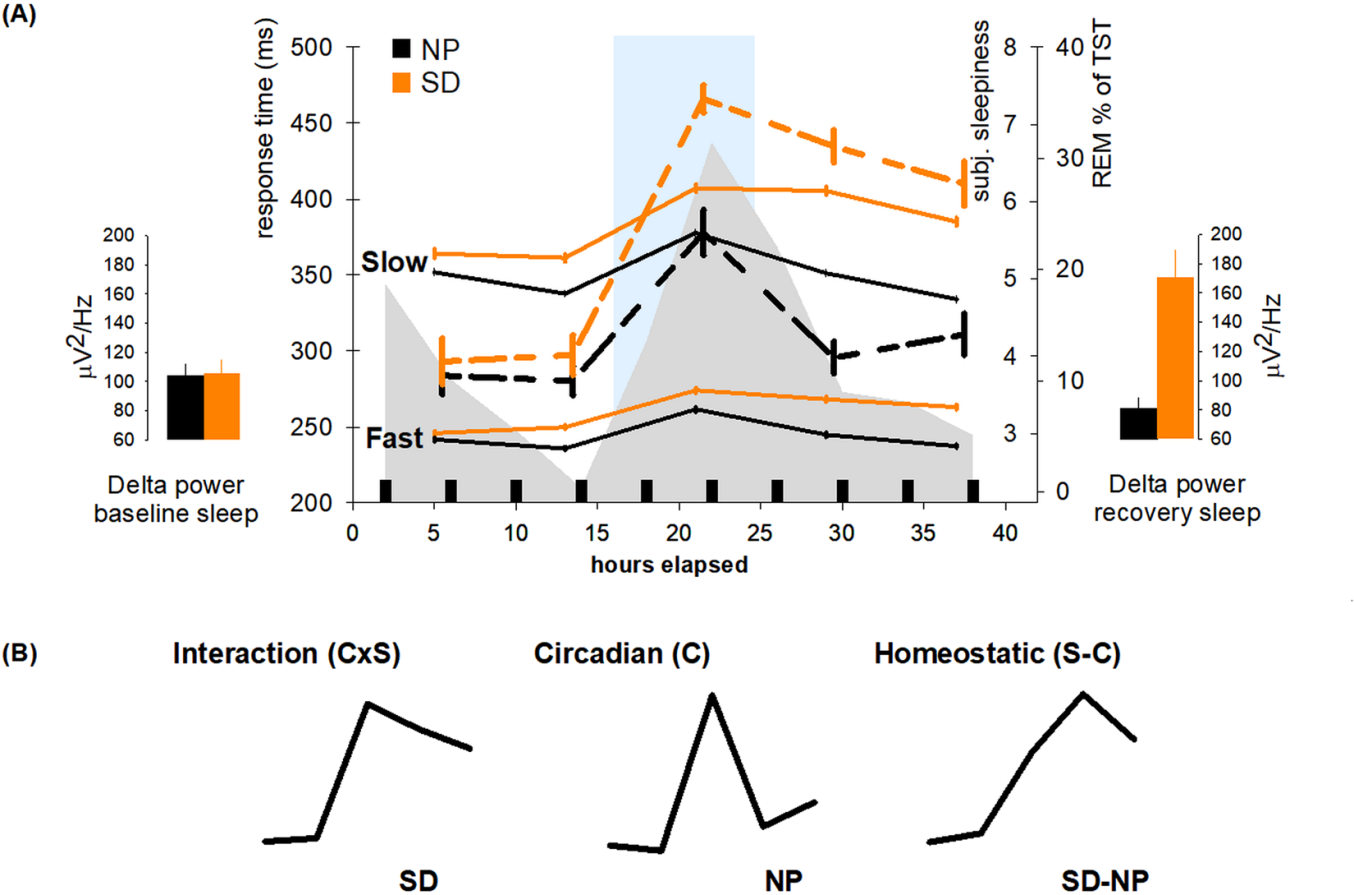
Sleepiness, vigilance, and sleep parameters during the study. (**A**) Orange: sleep deprivation (SD), black: nap protocol (NP). Left panel: Delta power during baseline sleep. Middle panel: Fast and slow reaction time (RT) courses (solid lines) and subjective sleepiness time courses (dashed lines), percentage of REM sleep per total sleep time (light grey area) during naps (small black squares). Error Bars represent standard errors of the mean. The blue area marks the biological night (16 to 24 h elapsed). Mean wake time (0 h elapsed) was 07:12 (±52 min). Right panel: Delta power during recovery sleep. (**B**) Illustration of sleepiness time courses used for contrast weighting in the fMRI analysis. Left panel: Values derived from SD, representing the interaction of the homeostatic and the circadian process (C x S), middle panel: values derived from the NP protocol, representing the circadian process (**C**), right panel: values derived from the difference between SD and NP scores (S-C). Units in (**B**) are arbitrary.

The interplay between circadian and homeostatic sleep-wake regulation mechanisms has been repeatedly tracked by assessing subjective sleepiness over the circadian cycle and under different sleep-wake schedules^10,11^. Subjective sleepiness is a red flag for an exhaustion of optimal daytime functioning^12^, however the cerebral mechanisms associated with its variation over the 24-h cycle remain to be established. Here, we investigated whether distinct vigilance-related brain activity profiles are temporally associated with the average fluctuation in subjective sleepiness over the 24-hour cycle. In order to tease the relative contribution of circadian and sleep homeostatic influences on sleepiness apart, vigilant attention and their cerebral correlates was assessed in 31 healthy participants in a balanced cross-over design that comprised a 40-h SD and a 40-h multiple nap protocol (NP). During both SD and NP, five functional magnetic resonance imaging (fMRI) sessions were individually scheduled at 5, 13, 21, 29, and 37 hours after each individuals’ habitual wake-up time (Fig. 1A). Participants (demographic data in Table 1) performed the Psychomotor Vigilance Task (PVT^13^) during these scan sessions.  Fig.1B highlights the time course of subjective sleepiness values across both protocols (group mean average assessed before and after each scan) matching the circadian and homeostatic slopes as predicted by the two-process model of sleep-wake regulation^14,15^. We explored BOLD activation modulation which was temporally linked to the time course of subjective sleepiness over the 24-h. We assumed this modulation to be amplified in task-related cortical regions under the sleep loss condition, particularly during nighttime^15^. Evidence suggests that optimal (fast) reaction times (RT) are differentially affected by circadian and homeostatic processes than non-optimal (slow) RTs^16^, and that sleep loss does not equally affect the cerebral correlates of poor and good performance^17^. We therefore distinguished between the time course of cerebral correlates underlying slow (>percentile 75) and fast (<percentile 25) RTs.

**Table 1:** Means (±STD) of demographic data and questionnaires.

*N* [m, f]	31 [14, 17]
Years of age	24.7 (3.3)
BMI [kg/m^2^]	22.2 (2.5)
Wake time [clock time]	07:12 (52 min)
Sleep time [clock time]	23:08 (53 min)
PSQI	3.13 (1.2)
ESS	4.2 (2.5)
MCTQ Sleep duration [h]	7.9 (0.8)
MCTQ MSFsc	4.35 (1.1)
MCTQ MSFsac	7.2 (2.5)
BDI-II	1.9 (2.2)

BMI = Body mass index, PSQI = Pittsburgh Sleep Quality Index^54^, ESS = Epworth Sleepiness Scale^55^, MCTQ = Munich Chronotype Questionnaire^56^, MSFsc = Mid sleep free days sleep corrected, MSFsac = Mid sleep free days sleep and age corrected, BDI = Becks Depression Inventory-II^57^. Wake and sleep times refer to baseline and recovery nights during the study.

## Results

### Time course of subjective sleepiness and vigilant attention

Subjective sleepiness was significantly higher under SD (Mean ± SEM: 5.7 ± 0.16) as compared to NP (Mean ± SEM: 4.3 ± 0.12; main effect of condition; (F (1, 270) = 123.64; *p* < 0.0001). The main effect of *session* (i.e., time of day) (F (4, 270) = 61.39; *p* < 0.0001) was significant, and the interaction of *condition* x *session* indicated that in both conditions, highest levels were reached during the biological night (16 to 24 h after scheduled wake up), but sleepiness significantly decreased during the second biological day in NP, while during SD, sleepiness levels remained high (Fig. 1A).

Vigilant attention performance differed depending on the sleep pressure *condition* (F (1, 570) = 178.71, *p* < 0.0001), *session* (F (4, 570) = 48.48, *p* < 0.0001), and *speed* (fast RTs, slow RTs) (F (1, 570) = 3117.58, *p* < 0.0001). A significant interaction of factors *condition* x *session* (F (4, 570) = 9.74, *p* < 0.0001) indicated that, although not significantly different during the first session (*p*_*corr*_ > 0.05), RTs were higher for the remainder of the SD than the NP protocol (Fig. 1A). In both conditions however, RTs were slowest at night, stabilized on the second day under SD and decreased again under NP. Post-hoc tests on the significant interaction of factors *condition x speed* (F (1, 570) = 22.94, *p* < 0.0001) revealed that slowest RTs were more affected by SD than fastest RTs (*p*_*corr*_ < 0.0001 for all sessions). The interaction *session x speed* was significant (F (4, 570) = 3, *p* = 0.018). Post-hoc tests for the slowest range showed that the decrease from session four (21 h into the protocol) to five (29 h into the protocol, see Fig. 1) was significant (*p*_*corr*_ < 0.001), while the decrease in the fast range at these times was not (*p*_*corr*_ > 0.05). The 3-way interaction *condition x session x speed* did not reach significance (F (4, 570) = 1.21, *p* = 0.304).

### Vigilance-related BOLD activation anchored to the time course of subjective sleepiness

We investigated whether the time course of vigilance-related brain activation followed the temporal profile of subjective sleepiness. For this purpose, we extracted z-scores of the group mean subjective sleepiness values for each session during SD and NP (temporal profiles depicted in Fig. 1B) and applied these scores as weighting factors of the session contrasts at the fixed effect level (see methods for details). We assessed whether there are brain regions under SD **(1)** in which BOLD activitation significantly followed the temporal profile of subjective sleepiness under SD (Fig. 1B, left panel), reflecting circadian and homeostatic interaction, and brain regions under SD **(2)** and NP **(3)** in which activation significantly followed the circadian profile of sleepiness observed under NP (Fig. 1B, middle panel). Finally, we also investigated brain regions in which BOLD activation over sessions followed a near-linear slope by removing circadian contribution during SD **(4)** via subtracting z-scored sleepiness values under SD from those under NP (Fig. 1B, right panel). Table 2 lists brain areas by sleep pressure condition and speed range (fast, slow) for these four contrasts of interest. All values are family wise error (FWE)-corrected (see methods for details).

**Table 2:** Task-related BOLD activation anchored to sleepiness time courses over the protocol by reaction time domain.

Brain area	Side	Z score	*P*_*FWE*_	x	y	z
**(1) Areas following the temporal profile of subjective sleepiness observed during SD, N** = **27**						
**Increase in Activation, T+**						
***Fast RT Range***						
Thalamus (dorso-medial)	R^†*^	4.48	0.007	8	−14	4
	L^†^	4.02	0.04	−6	−12	0
	L	4.01	0.04	−6	−16	4
Putamen	L^*^	4.30	0.015	−18	8	2
***Slow RT Range***						
n.s. on FWE level						
**Decrease in Activation**, T−						
***Fast RT Range***						
Postcentral gyrus	R^*†^	5.69	<0.001	38	−32	60
	R	5.38	0.003	48	−26	46
Inferior parietal lobe	L^†^	5.16	0.008	−32	−44	52
Lingual gyrus	R^*^	4.98	0.02	10	−56	0
***Slow RT Range***						
Lingual gyrus	R	5.02	0.01	20	−72	−2
**(2) Areas following a circadian profile of subjective sleepiness during SD, N** = **25**						
**Increase in Activation, T+ and Decrease in Activation, T−;**						
***Both RT Ranges***						
n.s. on FWE level						
**(3) Areas following a circadian profile of subjective sleepiness during NP, N** = **30**						
**Increase in Activation, T+ and Decrease in Activation, T−;**						
***Both RT Ranges***						
n.s. on FWE level						
**(4) Areas following the temporal profile of subjective sleepiness after removal of the circadian influence during SD, N** = **26**						
**Increase in Activation, T+**						
***Fast RT Range***						
Thalamus (dorso-medial)	R^†^	4.10	0.03	4	−12	2
	L^†^	4.51	0.007	−4	−12	0
	R^†^	4.21	0.02	6	−8	0
	L^†^	4.14	0.03	−6	−8	0
***Slow RT Range***						
n.s. FWE						
**Decrease in Activation**, T−						
***Fast RT Range***						
Precentral gyrus	R^†^	4.83	0.04	52	8	32
Postcentral gyrus	L^†^	4.97	0.02	−36	−44	60
Postcentral gyrus (area 2)	R^†^	5.6	0.001	46	−28	46
Postcentral gyrus (areas 1/3b)	R	5.37	0.004	60	−10	30
	R	5.04	0.015	54	−16	34
Postcentral gyrus (area 4a)	R^†^	5.58	0.001	40	−30	60
Superior parietal lobe	R^†^	4.96	0.022	24	−56	50
Superior/inferior parietal lobe	R	5.12	0.01	32	−48	56
Inferior parietal lobe	L^†^	5.05	0.015	−32	−44	52
Superior temporal gyrus	R	5.37	0.004	52	−42	12
	L	4.99	0.018	−52	4	−12
Middle temporal gyrus	R^†^	5.57	0.001	42	−68	18
	L	5.87	0.0003	−46	−64	4
	R	4.85	0.03	52	−60	12
Inferior temporal gyrus	R	5.74	0.0006	46	−58	−12
Superior occipital gyrus	L	5.34	0.004	−18	−86	36
	L	5.29	0.005	−22	−90	20
	R	5.13	0.01	22	−88	18
Middle occipital gyrus (V3)	L	5.72	0.0007	−24	−90	8
	R	4.92	0.025	36	−80	24
	R	5.00	0.018	30	−76	34
Inferior occipital gyrus (V4)	L	4.84	0.035	−36	−82	−6
	L	5.43	0.003	−30	−78	−10
Inferior occipital gyrus	L	5.02	0.016	−50	−74	−6
	L	4.85	0.034	−40	−70	−12
Lingual gyrus	R	5.10	0.011	18	−82	−4
Lingual gyrus/V1	L	5.06	0.01	−10	−58	−2
	R	5.50	0.002	8	−66	0
Fusiform gyrus	L	5.40	0.003	−20	−50	−12
	R	5.75	0.0006	34	−58	−14
Calcarine gyrus	R	5.62	0.001	14	−68	18
	R	5.45	0.002	6	−66	16
Cuneus	R	5.19	0.008	4	−74	18
***Slow RT Range***						
Inferior frontal gyrus	R	4.98	0.0150	54	34	12
Superior/Middle temporal gyrus	R	4.89	0.0220	58	−46	10
Cuneus	L	4.87	0.0230	−18	−84	2
	L	5.15	0.0072	−14	−82	20
Middle occipital gyrus	L	4.80	0.0315	−44	−76	−10
Area V2/BA 18	L^*^	5.03	0.0120	−16	−100	12
	L	4.99	0.0142	−18	−90	20
Inferior occipital lobe	L	5.13	0.0078	−34	−78	−8
Gyrus lingualis	R	5.08	0.0098	20	−74	−4
	R	4.79	0.0321	10	−68	−4
	L	5.02	0.0125	−16	−68	−10
Gyrus fusiformis	L	4.86	0.0248	−26	−72	−14
	R	4.88	0.0227	32	−76	−10
Gyurs calcarinus	R	4.84	0.0262	14	−72	18
	R	4.78	0.0336	22	−82	12

**Figure 2:**
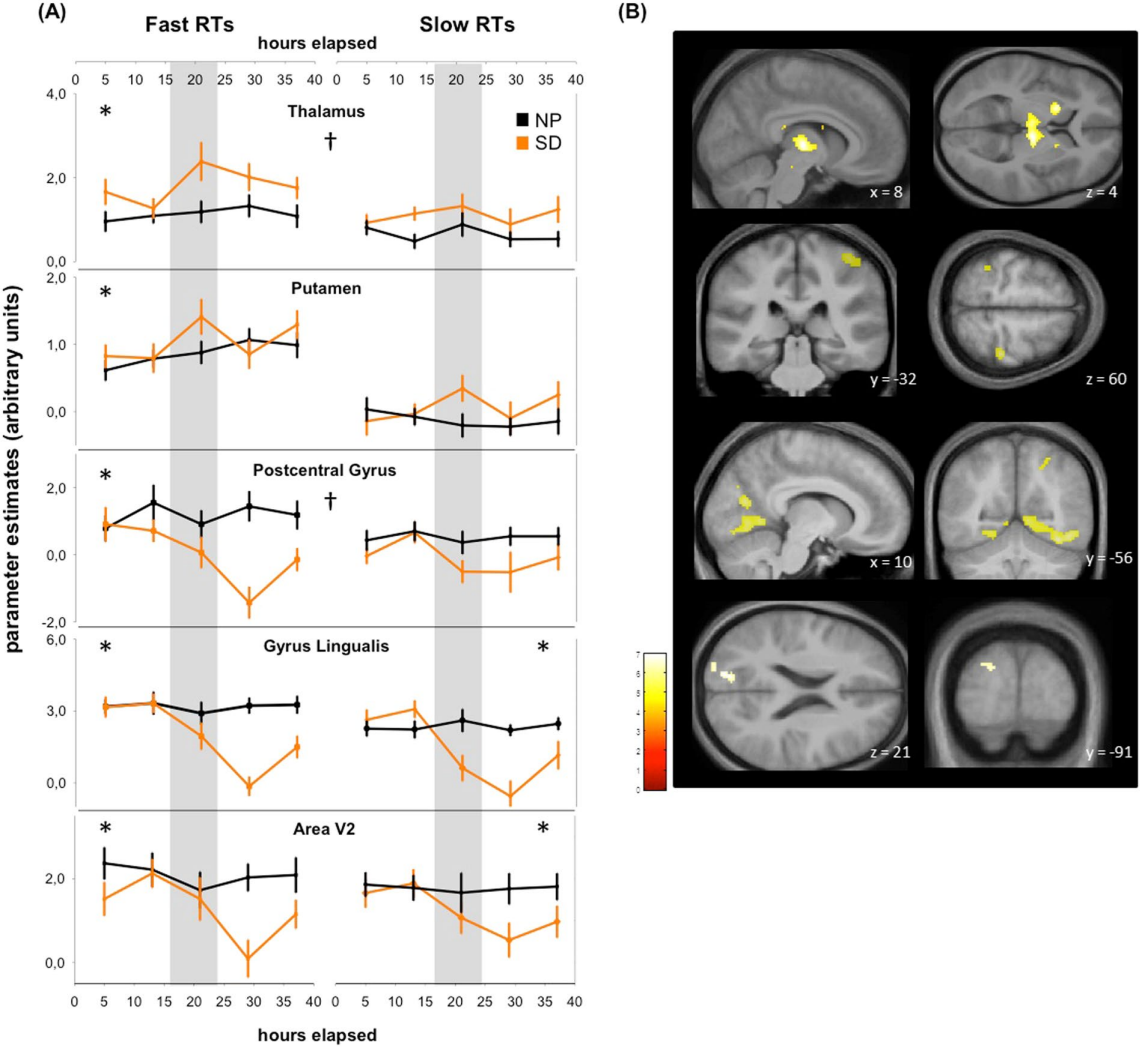
Time course of brain activation during both conditions underlying fast and slow RTs. (**A**) Parameter estimates of brain activity time courses during SD (orange lines), NP (black lines) for fast RTs (left panels) and slow RTs (right panels). Grey area covers the biological night (16 to 24 h awake). *Areas significantly following the temporal profile of subjective sleepiness during SD; ^†^significant difference between speed ranges. (**B**) Activity overlay on population mean structural image for corresponding brain areas, *p* < *0*.001 uncorrected display for illustration.

Data were thresholded at the voxel level, values of peak activity are reported. Coordinates (x, y, z) are expressed in mm in the Montreal Neurological Institute (MNI) space. *P*_*FWE*_: *p*-value after family-wise correction for multiple comparisons (FWE). C = circadian; S = homeostatic; SD = sleep deprivation, NP = nap protocol. R = right, L = left, B = bilateral. Areas marked with asterisks are shown in Fig. 2. ^†^denotes areas showing a significant difference between Fast and Slow RT range identified by exclusive masking. N.s. = not significant.

#### Fast RT range

Under SD, BOLD activation in the bilateral dorsomedial thalamus (Fig. 2) and the left putamen increased during the biological night (16 to 24 h after scheduled wake up) and partially stabilized on the second biological day, thereby following the time course predicted by subjective sleepiness under SD (contrast (1); T+, Table 2). In parallel, BOLD activation in a set of task-relevant cortical regions (right postcentral gyrus and lingual gyrus, left inferior parietal lobe, Table 2, Fig. 2, contrast (1); T−) significantly followed the reverse pattern, such that activity decreased throughout the night to reach minimal levels after 30 hours of prior wakefulness and stabilizing thereafter. The time course for these task-relevant cortical regions thus mirrored the time course of circadian sleepiness superimposed on homeostatic sleep pressure (Fig. 1B, left panel). Importantly, an even larger set of cortical activations showed a quasilinear decrease over the protocol that paralleled the time course of sleepiness under SD when the circadian impact was subtracted, (Table 2, Fig. 2, contrast (4), T−). Interestingly, we did not observe any regional BOLD activation that significantly followed sleepiness under NP, neither under high (contrast 2), nor under low sleep pressure (contrast 3) conditions, Fig. 1B, middle panel), suggesting that a circadian modulation in brain correlates which parallels the time course of subjective sleepiness can only be detected in interaction with sleep homeostatic pressure, that is accumulating sleep debt.

#### Slow RT range (>75 percentile)

As depicted in Table 2, we did not detect any region whose BOLD activation profile significantly paralleled sleepiness patterns for either the NP or SD condition. Only the lingual gyrus survived FWE-correction and mirrored the time course of subjective sleepiness under SD (Fig. 2). Furthermore, as for the fast RT domain, BOLD activation in a series of cortical regions presented a quasilinear decrease that mirrored the time course of sleepiness under SD, when the circadian impact (sleepiness under NP conditions) was subtracted (pattern depicted in Fig. 1B, right panel). These regions included the inferior frontal gyrus, a temporal and several occipital regions (see Table 2, contrast 4).

#### Fast vs Slow RT range

Activation in the bilateral dorso-medial thalamus was significantly more associated with the sleepiness time course for the fast compared to the slow RT domain (stronger nighttime increase, see Fig. 2). Furthermore, BOLD activation in a series of cortical regions was significantly more associated with the time course of subjective sleepiness for the fast compared to the slow RTs (regions denoted by † in Table 2). Note that all other areas listed in Table 2 also followed the respective sleepiness time courses when both slow and fast RT ranges were pooled.

### Link to electrophysiological and hormonal markers of sleep homeostasis and circadian rhythmicity

We included core physiological circadian and homeostatic markers as covariates to assess whether they affect the time course of vigilance-related BOLD activation anchored to the modulation of subjective sleepiness.

#### Electroencephalographic (EEG) slow-wave activity during Non-REM (NREM) sleep

**Figure 3:**
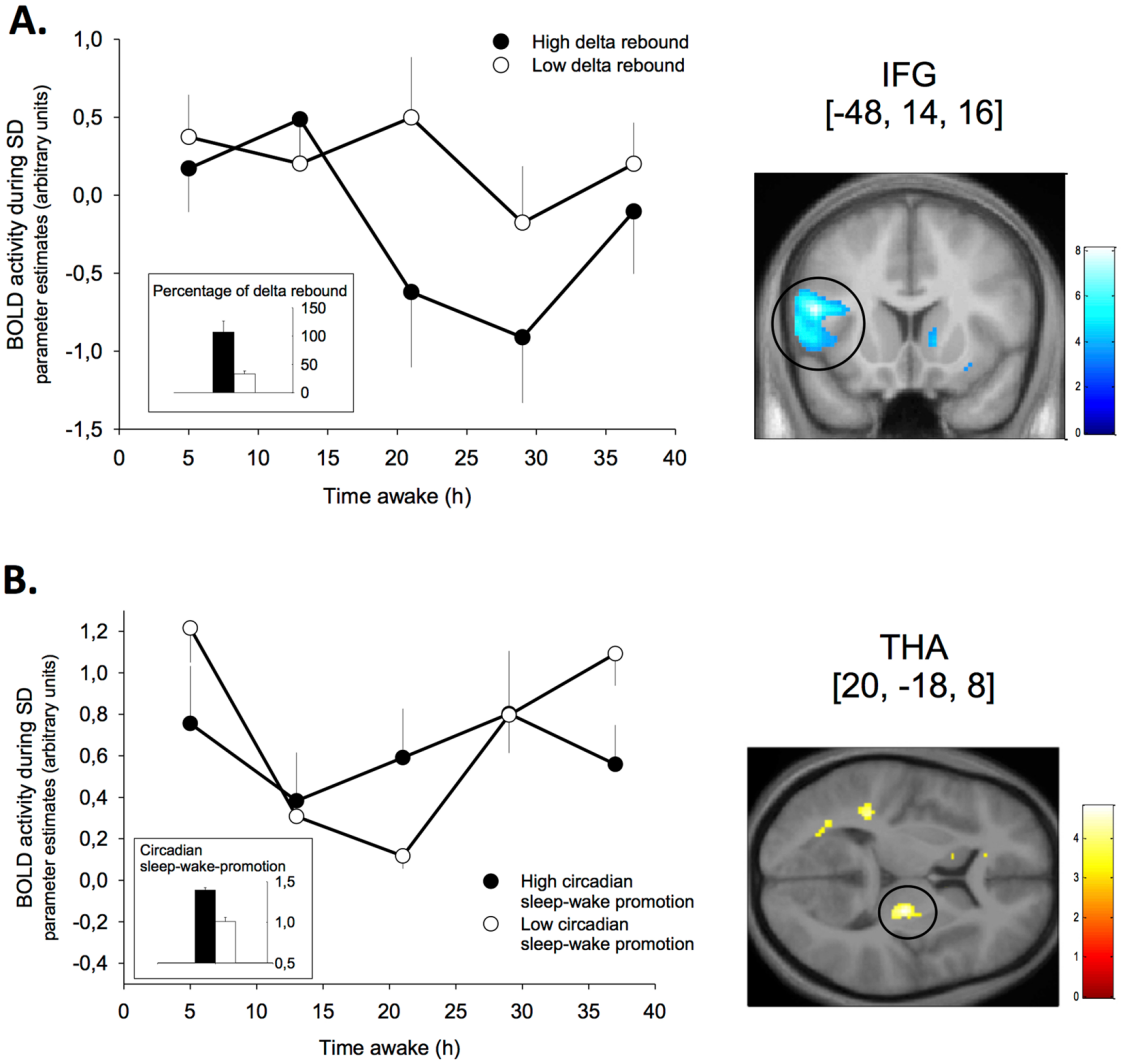
Homeostatic and circadian markers covary with BOLD activation time courses. **A.** Left panel: Time course of BOLD signals in the left inferior frontal gyrus (IFG) during sleep deprivation (SD) in participants with high (black circles) vs. low (white circles, median split for illustration) delta rebound (bar plot) during recovery sleep after 40 h SD. Right panel: Corresponding BOLD activation significantly covarying with delta rebound, overlay on group mean structural image, uncorrected display at *p* < 0.001 for illustration. **B.** Left panel: Time course of BOLD signal in the right thalamus (ventral lateral part) during SD in participants with high (black circles) vs. low (white circles, median split for illustration) circadian sleep-wake-promotion (bar plot). Right panel: Corresponding BOLD activation significantly covarying with circadian signal strength, overlay on group mean structural image, uncorrected display at *p* < 0.001 for illustration.

To assess accumulated sleep pressure through SD, we calculated the difference of NREM spectral power in the delta range (0.7–4 Hz) between the recovery night and the baseline night of the SD protocol^18^. We observed that participants with a higher EEG-derived delta activity rebound (e.g., experiencing higher sleep pressure levels) also had a greater nighttime BOLD activation decline in the left inferior frontal gyrus, the bilateral insula and a set of temporo-occipital regions under SD (Table 3, Fig. 3).

**Table 3:** Covariance of brain activity time courses with homeostatic and circadian markers.

Brain area	Side	Z score	*P*_*FWE*_	x	y	z
**Areas showing an inversed association to delta activity rebound along with subjective sleepiness in SD, N** = **26**						
***Fast RT***						
IFG (p. opercularis)	L	5.52	0.002	−48	14	16
IFG (p. triangularis)	L	4.94	0.026	−48	20	0
	L	4.98	0.022	−44	30	0
Insula	L	5.25	0.007	−34	22	−2
	R	5.01	0.0199	28	20	−12
Middle temporal gyrus	R	4.961	0.024	56	−18	−8
***Slow RT***						
Cerebellum	R	5.25	0.006	36	−44	−28
IFG (p. triangularis)	L	5.14	0.009	−54	18	20
Temporal pole	R	5.10	0.011	52	10	−20
Precuneus	L	4.95	0.021	−8	−58	32
Gyrus lingualis	R	4.87	0.028	10	−60	−6
Superior temporal gyrus	R	4.86	0.030	46	−26	−4
**Areas showing an inversed association to circadian sleep-wake-promotion along a circadian slope in SD, N** = **25**						
***Fast RT Range***						
Thalamus (ventral lateral part)	R	4.30	0.016	20	−18	8

Data were thresholded at the voxel level, values of peak activity are reported. Coordinates (x, y, z) are expressed in mm in the Montreal Neurological Institute (MNI) space. *P*_*FWE*_: *p*-value after family-wise correction for multiple comparisons (FWE). SD = sleep deprivation, IFG = inferior frontal gyrus, R = right, L = left, B = bilateral. N.s. = not significant. All ANCOVAS were tested with both RT ranges for positive (T+) and inversed (T−) associations, only significant results are listed.

#### Circadian amplitude and wake promoting strength

Our nap protocol allowed the extraction of sleep parameters at time windows of maximal circadian wake and sleep promotion. The inability to sleep (i.e., wakefulness during a sleep opportunity) during the so called “wake maintenance zone”^19^ is indicative for the strength of the circadian wake-promoting signal^3^. REM sleep shows a clear circadian modulation peaking in the late biological night to early morning hours, indicative of a circadian sleep-facilitating window^20^. Accordingly, the amount of REM correlated significantly with subjective sleepiness levels assessed before and after the nap (Spearman’s r = 0.4; *p* = 0.035). We estimated each individual’s strength of the circadian sleep- and wake-promotion by calculating the following composite score: we extracted each participant’s REM sleep peak (highest amount of REM sleep per total nap sleep time during the NP protocol, mean REM sleep time course see Fig. 1) summed up with the amount of wakefulness during the nap scheduled at the evening on the first day of the NP protocol. Circadian sleep-wake-promoting strength covaried with the circadian time course of a ventrolateral thalamic region, such that lower amplitude was associated with less pronounced nighttime increase in BOLD activation during fast RTs under SD (Fig. 3B).

## Discussion

Our data indicate that cortical resources required to sustain vigilant attention follow the time course of subjective sleepiness as predicted by the two-process model of sleep-wake regulation, with steep declines in task-related cortical responses once wakefulness is extended into the biological night. Moreover, the nighttime decrements in task-related cortical resources for vigilant attention were paralleled by increased thalamic and other subcortical responses, peaking when sleepiness is maximal. Intriguingly, circadian and sleep homeostatic modulations could only be traced at the cortical level with growing sleep debt (>16 hours), particularly during high as compared to low task engagement as indexed by RT speed.

Our data highlight the functional relevance of circadian and homeostatic regulation of neurobehavioral performance at the cerebral level and are in line with a recent report revealing a local modulation of human brain responses by circadian rhythmicity and sleep debt^4^. In accordance, we detected that BOLD activation in a set of task-related cortical brain areas traced the time course of the interaction between sleep pressure and circadian rhythmicity. Strong BOLD activation decreases in response to time spent awake were observed for all reported cortical areas. Furthermore, as Muto *et al*^4^, we observed relatively stable BOLD activation levels during the sessions scheduled during daytime, while a steep activation decline occurred in the late subjective night and early subjective morning, around the offset of melatonin production. Thus, at the cortical level we observed a profile clearly reflecting the combined influence of both the circadian and sleep homeostatic processes, while a different profile was observed for subcortical regions. In line with Muto *et al*^4^., we detected a significant circadian modulation in thalamic BOLD activation during sustained attention, which was temporally linked with the circadian melatonin profile. Importantly, our data provide further in-depth assessment of how these two systems impinge on the cerebral correlates of attentional resources. They indicate that circadian modulation at the cerebral level is only detectable under high sleep pressure conditions, particularly in subcortical structures, including the bilateral thalamus and the striatum, key players in arousal regulation^21^ and motor response control^22^. A recent meta-analysis on the effects of sleep loss on attention^9^ suggests that increased thalamic activity reflects a complex mutual interplay between the effects of sleep loss, which dampens arousal on one side, and the engagement in the task, which increases arousal on the other side. Our data show that thalamic activity peaks during the night, particularly for the fast reaction times, and lowers again during the subsequent day. The higher subcortical activity thus may provide a compensatory mechanism for the adverse circadian phase and high sleep pressure. Furthermore, the circadian system might channel the “need” for thalamic and other subcortical resources depending on sleep debt. Importantly, the nighttime increase in subcortical resources was specifically detected for the fastest RTs but not for slowest RT range during the task, suggesting an intermittent engagement to ensure optimal responses despite the challenging context of sleep loss and adverse circadian phase. The level of task engagement seems thus to contribute to the cerebral patterns bound to sleepiness observed in our data.

In parallel to subcortical activations peaking at night, we observed decreases in cortical activations (postcentral, inferior parietal and lingual gyrus) when wakefulness was extended beyond a classical waking day. This result is in line with earlier findings of sleep-loss related decrements in task-relevant cortical responses^4,23^. Interestingly, the activity decline was also observed and even further extended to mainly occipital and temporal regions when inspecting a near-linear homeostatic slope. Similarly, Muto *et al*^4^. observed an extensive cortical network to be modulated by a theoretical linear (homeostatic) slope. Furthermore, we observed that participants with greater slow wave activity rebound (i.e., higher sleep pressure levels) after SD had stronger decreases in activation in the IFG, insula, temporal and ventral occipital regions in both speed ranges, highlighting the impact of the sleep debt on cortical brain activity decreases. Interestingly, cortical brain activity in these regions declined more when optimally engaged in the task (fast responses), likely because of a combined influence of task-dependent local demands^24,25^ and time awake^26^. In the context of the PVT, the perception of visual stimuli putatively leads to a constant recruitment and disproportional use of occipital regions. It has been suggested that attenuated stimulus-related activation is due to compromised fronto-parietal top-down attention control and reduced sensitivity of primary sensory cortices to top-down or bottom-up inputs^27,28^. Similarly, reduced occipital cortex activation might result from reduced sensitivity of the visual cortex to sensory stimuli, with possible use-dependent effects^29–31^. Concomitantly, a local modulation of cerebral circadian phase may occur^4^, potentially in response to task-related requirements.

We did not detect any region where BOLD activation significantly followed a circadian slope under low sleep pressure conditions. It is well established that the amplitude of the circadian signal depends on sleep pressure^32^, such that nighttime decrements in neurobehavioral performance are amplified by increasing sleep pressure^33^. Our data thus extend previous reports suggesting that the impact of the circadian oscillator depends on the status of the sleep homeostat^15^, to the cerebral level. In line with this, we observed that participants with a lower circadian sleep-wake-promoting signal had a nightly dip in ventral lateral thalamic activity, whereas those with a stronger signal showed an increase during SD.

Our data shed light on cerebral mechanisms underlying the 24-h modulation in vigilant attention and provide an in-depth assessment of how circadian and homeostatic factors impinge on the brain’s attentional resources. This is of particular importance for shift workers and individuals suffering from jet lag, since they often show circadian misalignment and high sleep pressure levels due to a lack of sleep. Determinants of sleepiness are multifactorial, including factors such as life style and habits, stress, work schedules, but also sleep loss and circadian misalignment. Our data suggest that the two processes act on vigilant attention through selective mechanisms; with a homeostatic use of cortical resources and a circadian subcortical “rescuing” under sleep loss.

### Limitations

In this study, we focused on vigilant attention. As both the nature of cognitive domain and task complexity affect behavioral vulnerability to sleep loss, further studies are needed to investigate higher order cognitive function such as working memory under similar experimental conditions. In fact, whether a brain region shows an increase or a decrease in BOLD activation under SD depends on the investigated cognitive domain, and task complexity. Hence, different patterns may be elicited when investigating other tasks (e.g. working memory, decision making)^34^ under the same experimental conditions.

Our methodological approach was suitable to identify brain regions underlying vigilant attention, which parallel commonly observed 24-h sleepiness patterns assessed via a standardized questionnaire. However, it does not enable the observation of brain activity patterns which show different slopes or which depict activity alterations time-lagged to the course of sleepiness peaks and troughs. Nonetheless, insights on how sleepiness slopes are mirrored in vigilance and its brain correlates, may provide a basis for further translational research questions. Importantly, inter-individual variability needs to be taken into account before applying these findings in a clinical setting. Inspecting cerebral correlates along with individual sleepiness profiles might help understanding the vulnerability to sleep loss and circadian misalignment.

## Materials and Methods

### Participants

Thirty-three healthy young volunteers participated in the study, two participants dropped out by choice (final N = 31, mean age ± STD: 24.8 ± 3.3 years, 17 f, 14 m, Table 1). All participants were non-smokers and did not take any medication (except contraceptives for women). After completing several health and sleep quality questionnaires, participants underwent one night of polysomnography to exclude sleep disorders before participation. All further recruitment details and exclusion criteria are published in^35^. Women without contraceptives (two out of 17) participated during the luteal phase of their menstrual cycle. Participants were genotyped to control for vulnerability to sleep-loss regarding polymorphisms in PERIOD3 (rs57875989^36^; 15 PER35/5, 16 PER34/4) and adenosine deaminase (rs73598374^37^; 12 G/A-, and 19 G/G-allele carriers; frequency in this sample n.s., χ^2^ = 0.21). The study was approved by the local ethics committee (Ethikkomission beider Basel, EKBB, Switzerland), and all procedures conformed to the standards of the declaration of Helsinki. Participants gave their written informed consent.

### Procedure

Each volunteer completed two study blocks (56 h duration each) in the laboratory in a pseudo-randomized, balanced, crossover order. Both protocols were preceded by an 8-h baseline sleep episode at individual habitual bedtimes, the latter were held regular during seven days before study blocks (actimetry- and sleep log-controlled). In the NP, participants underwent 10 alternating cycles of 160 min of scheduled wakefulness and 80 min of scheduled sleep (i.e., naps) after habitual wake up times. In the SD, participants remained awake for 40 h after wake up at habitual times. Both blocks ended with a recovery night (minimum 8 h time in bed at habitual bedtimes). The combination of the two protocols allows an investigation of the circadian modulation in sleep and wake parameters, once under a continuous rise in homeostatic sleep pressure, and once under relatively low sleep pressure levels due to regular naps (see also e.g.,^10^). Data were collected under stringently controlled laboratory conditions. Participants remained in semi-recumbent posture position in bed at <8 lux light level during scheduled wakefulness, received regular light meals and had no time-of-day indication. Getting up was allowed for toilet visits at specific times throughout the protocol. During scheduled sleep episodes, light levels were at approximately zero lux and participants were in supine body posture. Except during fMRI acquisition, volunteers were continuously monitored by EEG. FMRI data were acquired at five time points (sessions), namely at 5, 13, 21, 29, and 37 h into both protocols (Fig. 1). The second and the last acquisition point (13 and 37 h awake) encompass the so-called wake-maintenance zone (average distance to DLMO ± STD = 51.2 min ± 66.5 min), whereas the nightly acquisition (21 h awake) covers the time window where melatonin secretion is maximal and where greatest deterioration in cognitive performance is usually observed^3,19,38^.

For previous publications based on this study see^35,39–44^.

### Behavior

#### Subjective sleepiness and vigilance

During both protocols, participants regularly rated their subjective sleepiness levels on the Karolinska Sleepiness Scale (KSS^45^). Here, we averaged samplings approx. 30 min before and after each task administration within the MR scanner, resulting in five data points (see Fig. 1). Vigilant attention was assessed with a psychomotor vigilance task (PVT) of 10 min duration at ten time points. Here, we focus on the five sessions which were performed within the MR scanner. The original PVT design^13^ was modified to suit fMRI admission. On a black screen, a white fixation cross was presented and at random intervals (2–10 sec), a millisecond counter started (clock event). Participants had to press a button to stop the counter as fast as possible with their dominant hand. We included null events (the fixation cross was replaced by a clock counter) at random in the task (25% of the trials, 2–10 sec duration). As performance feedback, the RT was displayed for one sec after each response. RTs >500 ms were classified as lapses. Errors of commission (i.e., random or anticipatory button presses) were not registered.

#### Behavioral data analysis

Group analyses of the sleepiness and PVT data were performed with the statistical package SAS (SAS Institute Inc., Cary, NC; version 9.3) with mixed-model repeated measures analysis of variance (PROC MIXED). *P* values were based on Kenward-Roger’s corrected degrees of freedom^46^. Post hoc contrasts were assessed with the LSMEANS statement and the Tukey-Kramer-correction for multiple comparisons was applied. PVT RTs were classified as follows for each participant in each session: RTs lower than the 25^th^ percentile (fast RTs), RTs higher than the 75^th^ percentile (slow RTs), RTs in the range between the 25^th^ and 75^th^ percentile (intermediate RT) and lapses (RTs >500 ms). Please note that here we focus on fast and slow RTs and do not consider intermediate RTs. We used the factors *condition* (NP vs. SD) and *session* (1–5), as well as *speed (fast vs. slow)* for the PVT analysis.

### Circadian and homeostatic markers

#### Circadian markers: REM-sleep, wakefulness, and melatonin

REM sleep shows a clear circadian modulation peaking in the late biological night to early biological morning hours^20^. Sleep efficiency however is lowest during the so called “wake maintenance zone”^19^ shortly before bedtime. The inability to sleep (i.e., wakefulness during a sleep opportunity) at this time is thus indicative for the circadian wake-promoting signal^3^. Here, we estimate the individual strength of the circadian sleep- and wake-promotion by considering these two markers in a composite score. To do so, we assessed each participant’s REM sleep peak (highest amount of REM sleep per total nap sleep time during the NP protocol, visually scored according to standard criteria^47^, details on polysomnography provided in^35^) and the amount of wakefulness during the nap scheduled at the evening on the first day of the NP protocol. Mean REM sleep time course is illustrated in Fig. 1. Circadian phase was assessed via salivary assays that were analysed for melatonin levels as described in a previous publication^35^. The individual melatonin amplitude was computed according to^48^.

#### Homeostatic marker: EEG Slow wave activity during NREM sleep

As a marker of homeostatic sleep pressure, we investigated the EEG slow wave activity (0.7–4 Hz) power^18^ during non-rapid-eye-movement (NREM) sleep (sum of sleep stages 1, 2, 3 and 4) in 8-hour baseline and recovery nights. Calculation was based on an automatic scoring algorithm (ASEEGA, Version 1.3, Physip^49^, France, accordance rate with manual scorings 82.9%,). After an automatic artefact rejection step, a fast Fourier transform with Hanning window for consecutive 30-sec epochs was used to calculate EEG power of the central derivation (CZ-PZ, see^43^ for details). To assess accumulated sleep pressure levels after SD, we calculated the difference of NREM sleep slow wave activity spectral power between the recovery night and the baseline night assessed in the SD protocol^10,50^. One participant was identified as an outlier (two interquartile ranges below the 25th percentile), and excluded from the respective analysis of covariance (see below).

### Functional MRI

#### FMRI data analysis

Functional MRI time series were acquired with a 3 Tesla MR Scanner (MAGNETOM Verio, Siemens Healthcare) with a standard twelve-channel head coil. A gradient echo-planar sequence using axial slice orientation (32 slices; voxel size: 3 × 3 × 3 mm³ with 0.75 mm interslice gap; matrix size 76 × 76 × 32; TR = 2200 ms; echo time = 32 ms; flip angle = 82°) was used to obtain multislice T2*-weighted fMRI images. For anatomical reference, structural T1-weighted images (sMRI) were acquired with a magnetization-prepared rapid gradient echo sequence (TR = 2000 ms, echo time = 3.37 ms, flip angle = 8°, field of view = 25.6 cm, matrix size = 256 × 256 × 176, voxel size = 1 × 1 × 1 mm^3^). 176 contiguous axial slices covering the entire brain were assessed in sagittal direction.

Data were analyzed with SPM8 (http://www.fil.ion.ucl.ac.uk) implemented in MATLAB 2014. Using standard SPM8 parameters, functional scans of each session were realigned using rigid body transformations, iteratively optimized to minimize the residual sum of squares between the first and each subsequent image separately for each session, and a mean realigned image was created. The mean functional image was coregistered to the structural T1-image using a rigid body transformation, optimized to maximize the normalized mutual information between the two images. Coregistration parameters were then applied to the realigned BOLD time series. The mapping from subject to MNI space was estimated from the structural image. The normalization parameters were subsequently applied to the individually coregistered BOLD times series, which were then spatially smoothed using an isotropic 8-mm full-width at half-maximum (FWHM) Gaussian kernel. The analysis of fMRI data based on a summary statistics approach was conducted in two serial steps accounting for fixed and random effects, respectively. At the fixed effect level, changes in brain responses were estimated for each subject at each voxel using a general linear model (GLM), including the responses to events associated with RTs lower than the 25th percentile (fast RTs), events associated with RTs higher than the 75th percentile (slow RTs), events linked to the RT-range between the 25th and 75th percentile (intermediate RTs) as well as lapses (RTs >500 ms). The average no. of fast events during NP resp. SD were 10.0 resp. 9.8 (session 1), 10.1 resp. 10.1 (session 2), 9.4 resp. 8.3 (session 3), 9.8 resp. 8.3 (session 4), 10.0 resp. 9.5 (session 5). The average no. of slow events during NP resp. SD were 9.4 resp. 9.8 (session 1), 9.3 resp. 9.1 (session 2) 9.3 resp. 9.5 (session 3), 9.5 resp 9.3 (session 4), 9.6 resp. 9.3 (session 5). The average no. of lapses (±STD) during NP resp. SD were 1.7 (±3.9) resp. 4.1 (±6.5) (session 1); 0.4 (±0.7) resp. 1.5 (±2.5) (session 2); 4.4 (±8.9) resp. 12.3 (±12.1) (session 3), 1.8 (±3.3) resp. 13.3 (±9.3) (session 4), 1.1 (±2.5) resp. 7.7 (±8.5) (session 5).

A time modulation regressor (first order polynomial) was added to account for time-on-task effects for each trial type in all sessions. Each event was modeled as a function representing its onset (i.e., at the time of presentation of stimulus). The ensuing vectors were convolved with the canonical hemodynamic response function (HRF) and used as regressors in the individual design matrix. Six movement parameters accounting for translation and rotation, derived from realignment of the functional volumes, were included as regressors of no interest.

Regions of Interest (ROIs) were identified based on previous findings: In a recent meta analysis, the thalamus was identified as the only brain region consistently showing an increase in activation in different attention tasks under SD^9^, and therefore chosen as a ROI here. Further, the basal ganglia were previously shown to be implicated in motor speed tasks such as the PVT^17^, while the hypothalamus was set as a ROI because of its implication in circadian wake promotion^51^. For the ROI analysis in SPM8, we used the predefined masks implemented in the MARINA tool for the basal ganglia and the thalamus (further information and references available on http://www.bion.de/eng/MARINA.php), and around coordinates (6 mm radius) from the literature for the hypothalamus^52^. Finally, MRIcron (http://www.mccauslandcenter.sc.edu/mricro/mricron/) was used to combine the ROIs into a single mask.

Our main aim was to assess BOLD activation modulations over sessions, anchored to the average sleepiness values of our participant group. We extracted z-scores of the group mean subjective sleepiness values evaluated during SD and NP (time courses depicted in Fig. 1B) and used them to build a parametric contrast at the fixed effect level. This analysis allows us to investigate whether over sessions, BOLD activation significantly follows the temporal profile of subjective sleepiness during SD (contrast (1) in Table 2), reflecting the interacting pattern between homeostatic sleep pressure rise and circadian sleep-wake promotion. Z-scored group mean KSS values were extracted from the SD condition and used to build the parametric contrasts. We further investigated whether BOLD activation significantly follows a circadian pattern of sleepiness under high (contrast (2) in Table 2) and low (contrast (3) in Table 2) sleep pressure conditions by using z-scores of the group mean subjective sleepiness values evaluated under NP (time courses depicted in Fig. 1B). Finally, in a last contrast, we assessed whether there are regions which BOLD activation adopts a near-linear homeostatic slope during SD by subtracting z-scored sleepiness values under SD from those under the NP condition (contrast (4) in Table 2). We conceptualize contrast (4) as near-linear homeostatic slope, because a subtraction of SD and NP should not be interpreted as a pure homeostatic impact since these processes have been shown to interact in a non-additive manner^15^.

In the second level analyses, we applied one sample t-tests on the parametric contrasts described above to proceed to statistical inference. Further, at the between-subject level, we included the above described markers of circadian sleep-wake-promotion and homeostatic (slow wave activity rebound after the SD) markers as covariates to investigate whether they affect the time course of vigilance-related BOLD activation anchored to the modulation of subjective sleepiness (assessed by the parametric contrasts described above).

Statistical inferences were performed at a threshold of *p* = 0.05 after correction for multiple comparison (family-wise-error, FWE-correction) either at whole brain-level or over a set of a-priori defined regions of interest (ROIs). At the subcortical level, we expected BOLD activation changes in the thalamus^53^, basal ganglia^17^ and hypothalamic regions^51^. Differences between speed ranges were assessed by exclusive masking at *p* = 0.05 of contrasts of interests from slowest and fastest RT before applying the described correction for multiple comparison. Main effects of speed over both conditions were tested for regions that were showing any significant slope.

### Missing imaging data

A total of 11 datasets out of 300 (3.6%) were missing due to technical problems. Thereof, three participants had two sessions missing (20%); five participants had one session missing (10%). We handled missings as follows: whenever slopes were statistically similar to those without missings shown in Fig. 1B (significant cross-correlation at lag 0 of slope with and without missings), data were included in contrasts where appropriate. The respective N is indicated for each contrast in the results tables.

### Data availability

The datasets analysed during this study are available from the corresponding author upon reasonable request.
