# Author Correction: Human brain patterns underlying vigilant attention: impact of sleep debt, circadian phase and attentional engagement

**DOI:** 10.1038/s41598-019-48856-0

**Published:** 2019-08-22

**Authors:** Micheline Maire, Carolin F. Reichert, Virginie Gabel, Antoine U. Viola, Christophe Phillips, Christian Berthomier, Stefan Borgwardt, Christian Cajochen, Christina Schmidt

**Affiliations:** 1 Centre for Chronobiology, Psychiatric Hospital of the University of Basel, Basel, Switzerland; 2 Institute of Primary Health Care (BIHAM), University of Bern, Bern, Switzerland; 3 Transfaculty Research Platform Molecular and Cognitive Neurosciences, University of Basel, Basel, Switzerland; 4 GIGA-CRC In Vivo Imaging, University of Liège, Liège, Belgium; 5 Medical Image Analysis Center, University Hospital of Basel, Basel, Switzerland; 6 Department of Psychiatry, University Hospital of Basel, Basel, Switzerland; 7 PHYSIP SA, Paris, France; 8 PPRS, Paris, France

Correction to: *Scientific Reports* 10.1038/s41598-017-17022-9, published online 17 January 2018

The original version of this Article contained errors in the spelling of the authors Micheline Maire, Carolin F. Reichert, Virginie Gabel, Antoine U. Viola, Christophe Phillips, Christian Berthomier, Stefan Borgwardt, Christian Cajochen & Christina Schmidt which were incorrectly given as M. Maire, C. F. Reichert, V. Gabel, A. U. Viola, C. Phillips, C. Berthomier, S. Borgwardt, C. Cajochen & C. Schmidt respectively.

These errors have now been corrected in the PDF and HTML versions of the Article.

